# Dose–Response Effect of Vibratory Stimulus on Synaptic and Muscle Plasticity in a Middle-Aged Murine Model

**DOI:** 10.3389/fphys.2021.678449

**Published:** 2021-06-11

**Authors:** Ida Cariati, Roberto Bonanni, Giuseppe Annino, Manuel Scimeca, Elena Bonanno, Giovanna D’Arcangelo, Virginia Tancredi

**Affiliations:** ^1^Ph.D. in Medical-Surgical Biotechnologies and Translational Medicine, Department of Clinical Sciences and Translational Medicine, “Tor Vergata” University of Rome, Rome, Italy; ^2^Department of Systems Medicine, “Tor Vergata” University of Rome, Rome, Italy; ^3^Centre of Space Bio-Medicine, “Tor Vergata” University of Rome, Rome, Italy; ^4^Department of Biomedicine and Prevention, “Tor Vergata” University of Rome, Rome, Italy; ^5^Department of Experimental Medicine, “Tor Vergata” University of Rome, Rome, Italy; ^6^“Diagnostica Medica” and “Villa dei Platani”, Neuromed Group, Avellino, Italy

**Keywords:** whole body vibration, synaptic plasticity, hippocampus, muscle plasticity, mechanical vibration

## Abstract

Whole body vibration plays a central role in many work categories and can represent a health risk to the musculoskeletal system and peripheral nervous system. However, studies in animal and human models have shown that vibratory training, experimentally and/or therapeutically induced, can exert beneficial effects on the whole body, as well as improve brain functioning and reduce cognitive decline related to the aging process. Since the effects of vibratory training depend on several factors, such as vibration frequency and vibration exposure time, in this work, we investigated whether the application of three different vibratory protocols could modulate synaptic and muscle plasticity in a middle-aged murine model, counteracting the onset of early symptoms linked to the aging process. To this end, we performed *in vitro* electrophysiological recordings of the field potential in the CA1 region of mouse hippocampal slices, as well as histomorphometric and ultrastructural analysis of muscle tissue by optic and transmission electron microscopy, respectively. Our results showed that protocols characterized by a low vibration frequency and/or a longer recovery time exert positive effects at both hippocampal and muscular level, and that these effects improve significantly by varying both parameters, with an action comparable with a dose–response effect. Thus, we suggested that vibratory training may be an effective strategy to counteract cognitive impairment, which is already present in the early stages of the aging process, and the onset of sarcopenia, which is closely related to a sedentary lifestyle. Future studies are needed to understand the underlying molecular mechanisms and to determine an optimal vibratory training protocol.

## Introduction

Long-term exposure to a whole body vibration (WBV) stimulus was studied to obtain information about the increased health risk to which certain classes of workers are exposed daily (Bovenzi, 2015; Griffin, 2015; Krajnak, 2018). It has been reported that exposure to mechanical vibration, over prolonged periods of time, increases the risk to the health of the spinal column and peripheral nervous system, as well affects, although to a lesser extent, the digestive system, the female reproductive system, and the vestibular system (Bovenzi, 2006; Bovenzi et al., 2011; Beard and Griffin, 2016; Stoyneva et al., 2016; Charles et al., 2018; Yilmaz and Ila, 2019).

However, in the last decades research has shown that mechanical vibration represents a strong stimulus for the entire organism and especially for the neuromuscular system. Not surprisingly, WBV is now considered a simple and effective training method to increase physical performance and is used for a variety of purposes, including not only training of elite athletes, but also treatment for osteoporosis and chronic low back pain as well as neurological rehabilitation to reduce spasticity (Bosco et al., 1999a, b; Rittweger et al., 2002; del Pozo-Cruz et al., 2011; Prioreschi et al., 2012; Chan et al., 2013; Annino et al., 2019). Although most scientists agree that acute and chronic exposure to mechanical vibration induces significant improvements in muscle strength, some authors have not found significant effects, probably due to the use of different protocols (de Ruiter et al., 2003; Cochrane et al., 2004; Cochrane and Stannard, 2005; Annino et al., 2007; Di Giminiani et al., 2009; Jordan et al., 2010). In fact, it is known that neuromuscular performance can be influenced by several factors, such as the type of application, amplitude, frequency, and time of vibration exposure (Luo et al., 2005). For example, it has been shown that the application of high-intensity, short-duration mechanical vibrations contributes to the optimal maintenance of muscle mass and strength, promoting healthy muscles, bones, and joints, even in older people (Bogaerts et al., 2007, 2009; Ebid et al., 2012; Prioreschi et al., 2012; Annino et al., 2017).

The effects of WBV training on the central nervous system are not yet fully understood. To date, exposure to low-frequency mechanical vibration (40 Hz) has been shown to improve brain function and prevent post-ischemic cognitive decline (Raval et al., 2018). It has also been reported that daily WBV training lasting 10 min, five times a week for 5 weeks, at a frequency of 30 Hz, improves both motor performance in 15-week-old male mice and response to cognitive tests in healthy human subjects over 40 years old (Boerema et al., 2018). Furthermore, it was observed that the combination of WBV training with squat exercises induces an increase in plasma levels of Brain-derived neurotrophic factor (BDNF) in elderly women with knee osteoarthritis, suggesting a role of WBV in neuromuscular function and adaptation (Simão et al., 2019). Indeed, it is known that mechanical vibrations stimulate the production of neurotrophins, which act both by regulating the natural cell death of neurons during development and by stimulating the survival of different neuronal populations *in vitro* (Huang and Reichardt, 2001).

Based on these considerations, in our previous work we evaluated the effects of three WBV training protocols, differing in vibration frequency and vibration exposure time, on synaptic plasticity in an experimental mouse model, by means of *in vitro* extracellular recordings in hippocampal slices from 4- to 24-month-old mice (Cariati et al., 2021). It was previously suggested that synaptic plasticity is positively affected by WBV training, both in rodents (Manthou et al., 2017) and in humans (Fuermaier et al., 2014; Regterschot et al., 2014), probably through the involvement of sensory stimulation leading to cognitive enhancement. In agreement with these studies, our results showed that WBV training can modulate synaptic plasticity differently, depending on the protocol used, and that the best effects are related to the training protocol characterized by a low vibration frequency and a longer recovery time. Surprisingly, 24-month-old mice exposed to mechanical vibrations showed full recovery of cognitive capacity (Cariati et al., 2021), suggesting that vibratory training may be considered an important factor in protecting and/or preventing the development of age-related cognitive disorders, which in turn is closely related to synaptic plasticity (Weber et al., 2015; De Domenico et al., 2017). It is well known that aging is a biological process associated with physiological cognitive decline, as it can impair quality of life and cause deficits in memory and learning processes (Bettio et al., 2017).

Although most studies aimed at understanding the mechanisms underlying the aging process have focused on older people, there is strong scientific evidence for cognitive decline in middle age. In this regard, Boyer et al. recently reported that middle-aged mice (9-month-old) show significant behavioral changes in terms of social interactions compared with young mice (3-month-old). This suggests that although cognitive and sensory function appears to be preserved in 9-month-old mice, some neuronal circuits may be particularly sensitive to the aging process (Boyer et al., 2019). The levels of important regulatory proteins for processes such as neurogenesis, learning and memory (BDNF; cAMP response element-binding protein, p-CREB; neurite-promoting factor, NPF) were also found to be significantly reduced in the dentate gyrus and in the CA1 and CA3 subfields of the hippocampus in middle-aged rats (Hattiangady et al., 2005). Similarly, Shoji et al. (2016) reported that middle-aged rats (8–12-month-old) show impaired learning and memory processes, indicating that the cognitive decline that characterizes the aging process may be caused by proteomic alterations in the hippocampus. Finally, Adler et al. (2019) observed altered circadian rhythms in middle-aged mice (11–13-month-old), suggesting that the aging process may significantly affect the coordination of hippocampal functions, including energy metabolism, neurotransmission, and synaptic plasticity.

Based on this evidence, in the present work we evaluated the effects of three WBV protocols, differing in vibration frequency and vibration exposure time, on synaptic and muscle plasticity in a middle-aged murine model, to test whether WBV training could represent a tool to prevent and/or counteract the onset of the first symptoms of cognitive and muscle decline characterizing the aging process.

## Materials and Methods

### Animals

Twenty 12-month-old male mice, belonging to the strain wild type BALB/c mice, were used according to the procedures established by the European Union Council Directive 2010/63/EU for animal experiments (Louhimies, 2002). All the experimental protocols were approved by the Italian Ministry of Public Health (authorization n. 86/2018-PR). The animals were divided into five groups: three groups of trained mice (four mice per group), each is subjected to a different vibratory training protocol, and two control groups (four mice per group). For the latter, we used a control group (CTRL WBV) with mice subjected to the same regimen of placement on the box in the platform, the same environmental exposure including motor sounds, but not exposed to vibratory training; and another control group (CTRL SED) including sedentary mice not subjected to any type of training. All experimental animals were kept under the same housing conditions and diet.

Resident veterinarians and experimenters daily monitored the housing conditions of animals, in terms of air/min replacement, humidity, and temperature, providing constant environmental enrichment of the cages with food and water. All experimental animals were kept in the same housing conditions and on the same diet and were housed individually. In addition, during the training period, physical condition of the animals was evaluated, considering weight, coat and skin condition, and body functions.

### Training Protocols

A vibrating platform (Power Club, Vigarano Mainarda, 44049 FE, Italy) was used to subject animals to WBV. It has a power supply of 220 V and a total maximum electrical power of 0.12 kW. The function generator generates a sinusoidal control signal at the frequencies used: 45 Hz, with an acceleration of 2 *g* and a shift of 1.5 mm, and 90 Hz, with an acceleration of 2.8 *g* and a shift of 1.1 mm.

Vibration training was conducted using three protocols (A, B, and C), which differ in terms of vibration frequency and exposure time to vibration (Cariati et al., 2021). The A and B protocols consisted of five series (consecutive number of repetitions) of 3 min each, interspersed with 1 min of recovery. The C protocol consisted of three series of 2 min and 30 s each, with a recovery period of equal duration. The vibration frequency used was 90 Hz for the A protocol and 45 Hz for B and C protocols (Cariati et al., 2021). The animals underwent a total of 36 days of activity; specifically, each group of mice was trained for 12 weeks, on a 3-weekly basis. The animals were raised on a light: dark cycle of 12:12 h, and training was carried out in the morning, between 10 and 11 am.

### Electrophysiological Recordings in Mouse Hippocampal Slices

At the end of the training period, the mice were sacrificed as previously reported (Palmieri et al., 2019), making every effort to reduce the number of animals used and their suffering. The obtained hippocampal slices (450 μm thick) were transferred to an interface tissue chamber constantly perfused by a flow of 1.2 ml/min of artificial cerebral spinal fluid and humidified gas (95% O_2_, 5% CO_2_) at 32–34°C (pH 7.4). The extracellular recordings of population spike (PS), that indicates the electrical activity of a neurons population, were made according to procedures previously indicated (D’Arcangelo et al., 2017). After recording stable signals (15–20 min), a tetanic stimulation (100 Hz, 1 s) was delivered to induce the Long-term Potentiation (LTP) at the same stimulus intensity used for the baseline responses. Signals were acquired, digitized, and stored using a personal computer with standard acquisition software (Axon Instruments, Foster City, CA, United States). Signal was fed to a computer interface (Digidata 1440A, Axon Instruments, Foster City, CA, United States) for subsequent analysis with the software PCLAMP10 (Axon Instruments, Foster City, CA, United States).

### Histomorphometric Analysis

Immediately after sacrifice, muscle tissue was collected from the left side of the animals, taking samples from the quadriceps muscle. Then, muscle biopsies were fixed in 4% paraformaldehyde for 24 h and paraffin embedded. Three-micrometer thick sections were stained with hematoxylin and eosin (H&E) and the pathological evaluation was performed blindly by two pathologists. To assess the diameter of muscle fibers, 150 fibers per biopsy were evaluated. To calculate muscle fiber diameters, H&E slides were scanned at 10 × magnification by Nikon upright microscope ECLIPSE Ci-S/(Nikon Corp. Japan). Muscle fiber diameters were identified by a pathologist for each muscle biopsy and analyzed by Software NIS-ELEMENTS (5.30.01; Laboratory Imaging; Praha; Czech).

### Transmission Electron Microscopy

One mm^3^ of muscle tissue from left quadriceps biopsies were fixed in 4% paraformaldehyde and post-fixed in 2% osmium tetroxide (Cariati et al., 2020). After washing with 0.1 M phosphate buffer, the sample was dehydrated by a series of incubations in 30, 50, and 70% ethanol. Dehydration was continued by incubation steps in 95% ethanol, absolute ethanol, and propylene oxide, after which samples were embedded in Epon (Agar Scientific, Stansted, Essex CM24 8GF, United Kingdom; Palmieri et al., 2019). Ultra-thin sections, 80 nm were mounted on copper grids and examined with a transmission electron microscope (Model JEM-1400 series 120 kV, JEOL USA, Inc. 11 Dearborn Road Peabody, MA, United States; DigitalMicrograph^TM^ Software).

### Statistical Analysis

Statistical analysis was performed using GraphPad Prism 8 Software (Prism 8.0.1, La Jolla, CA, United States). For electrophysiological experiments, data were expressed as mean ± SEM, and *n* represents the number of slices analyzed. For histomorphometric analysis, data were expressed as mean ± SEM, and *n* represents the number of fibers analyzed. Data were compared with two-way ANOVA and Dunnett’s multiple comparison test and were considered significantly different if *p* < 0.05.

## Results

### Effects of Different Training Protocols on Synaptic Plasticity

**FIGURE 1:**
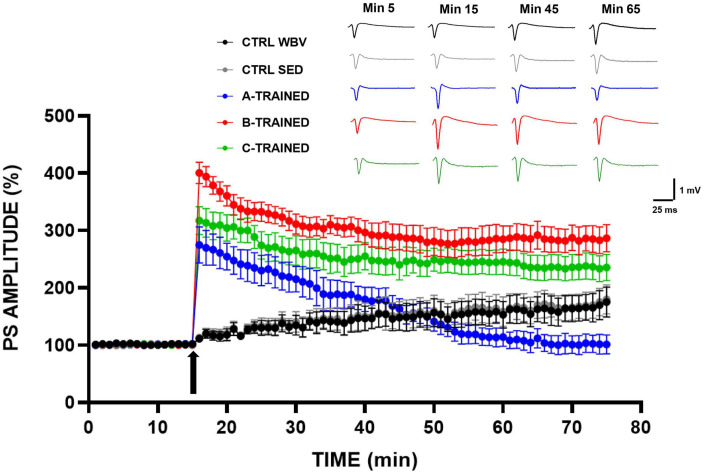
Synaptic plasticity in CA1 hippocampal subfield of old mice.% population spike (PS) amplitude as a function of time after HFS, applied at time t = 15 (arrow), is shown in CTRL WBV (black line, *n* = 7), in CTRL SED (gray line, *n* = 7), in A-TRAINED (blue line, *n* = 7), in B-TRAINED (red line, *n* = 8), and in C-TRAINED (green line, *n* = 9) mice slices. The insert shows representative recordings obtained from slices of each experimental group; the curves of each group refer to population spike at times 5, 15, 45, and 65 min.

The effects of the three types of vibratory training on synaptic plasticity were analyzed in the CA1 region of mice hippocampal slices and the results are shown in Figure 1. It is evident that in the three groups of trained mice, a tetanic stimulus induced at the 15 min of recording caused a sustained increase in PS amplitude values, which were significantly higher than in both control groups. These results agree with the data reported in our previous work (De Domenico et al., 2017), in which we observed that the synaptic plasticity of 12-month-old sedentary mice was characterized by a total block in the LTP induction phase and a slight recovery in the LTP maintenance phase. On the contrary, as regard the trained groups, we recorded PS amplitude values for the LTP maintenance phase that were different according to the experimental group considered. Specifically, B and C training protocols appeared to positively modulate synaptic plasticity throughout the electrophysiological recording time, since PS amplitude values remained significantly higher than those of the other experimental groups. The A training protocol, conversely, induced a drastic reduction in synaptic plasticity from about the 45th minute of recording, with PS amplitude values significantly lower than those of the other experimental groups.

The PS amplitude values recorded for each group of mice at various times are reported in Table 1, where the values of statistical significance are also shown.

**TABLE 1:** Percentage of population spike (PS) amplitude values recorded in the CA1 region of hippocampal slices from control and trained groups at different times.

**TIME (min)**	**CTRL WBV (PS% Amplitude)**	**CTRL SED (PS% Amplitude)**	**A-TRAINED (PS% Amplitude)**	**B-TRAINE D (PS% Amplitude)**	**C-TRAINED (PS% Amplitude)**	**Significance**
5	101.2 ± 0.7	102.4 ± 0.8	101.7 ± 0.8	102.0 ± 0.9	101.3 ± 0.7	**/**
15	111.0 ± 4.1	113.2 ± 3.9	274.6 ± 31.6	400.6 ± 17.8	317.2 ± 24.3	CTRL WBV and CTRL SED vs A-TRAINED, B-TRAINED and C-TRAINED, ****p < 0.0001
45	148.0 ± 22.3	151.2 ± 24.7	163.4 ± 17.9	288.8 ± 22.3	240.1 ± 26.4	CTRL WBV and CTRL SED vs B-TRAINED and C-TRAINED, ***p < 0.001
75	175.3 ± 26.1	179.9 ± 25.4	101.1 ± 16.7	286.6 ± 23.6	235.6 ± 22.8	CTRL WBV and CTRL SED vs B-TRAINED and C-TRAINED, ***p < 0.001; CTRL WBV and CTRL SED vs A-TRAINED, **p < 0.01

### Muscle Plasticity Following Vibratory Training

**FIGURE 2:**
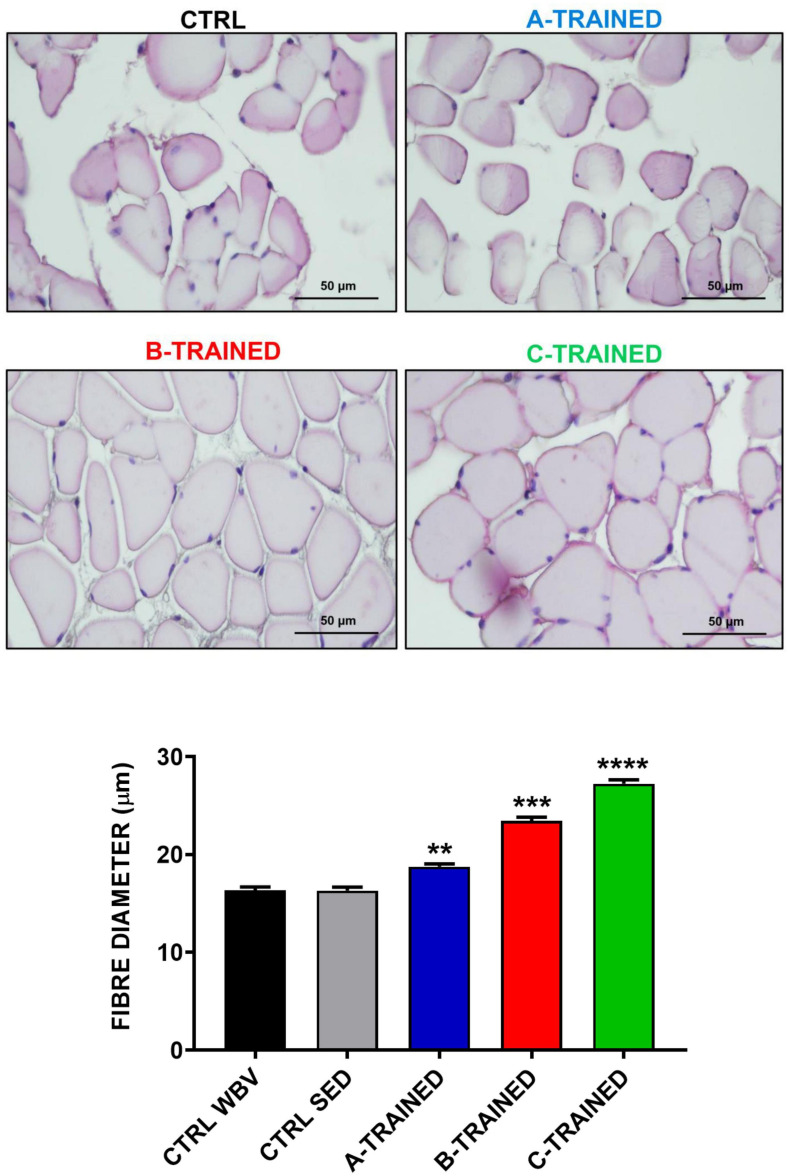
Evaluation of muscle fiber diameter following whole body vibration (WBV) training by histomorphometric analysis. The upper four panels show a representative section of muscle tissue from each experimental group (scale bar, 50 μm). The lower panel shows the mean value of the muscle fibers diameter in CTRL WBV (black bar, *n* = 150), in CTRL SED (gray bar, *n* = 150), in A-TRAINED (blue bar, *n* = 150), in B-TRAINED (red bar, *n* = 150), and in C-TRAINED (green bar, *n* = 150) groups. Note that a significant increase in muscle fiber diameter was found in the trained groups compared with the control groups (CTRL WBV and CTRL SED vs A-TRAINED, **p < 0.01; CTRL WBV and CTRL SED vs B-TRAINED, ***p < 0.001; and CTRL WBV and CTRL SED vs C-TRAINED, ****p < 0.0001).

Muscle plasticity after exposure to WBV training was assessed by histomorphometric analysis of the muscle tissue of each experimental group. As shown in Figure 2, the muscle fiber diameter values of both control groups (CTRL WBV 16.3 ± 0.4; CTRL SED 16.3 ± 0.3) were significantly lower than those of the three trained groups (A-TRAINED 18.8 ± 0.3,^∗∗^*p* < 0.01; B-TRAINED 23.4 ± 0.4, ^∗∗∗^*p* < 0.001; and C-TRAINED 27.2 ± 0.4,^****^p < 0.0001). Noteworthy, of the three groups undergoing WBV training, the mice trained with the C protocol had the highest muscle fiber diameter values.

**FIGURE 3:**
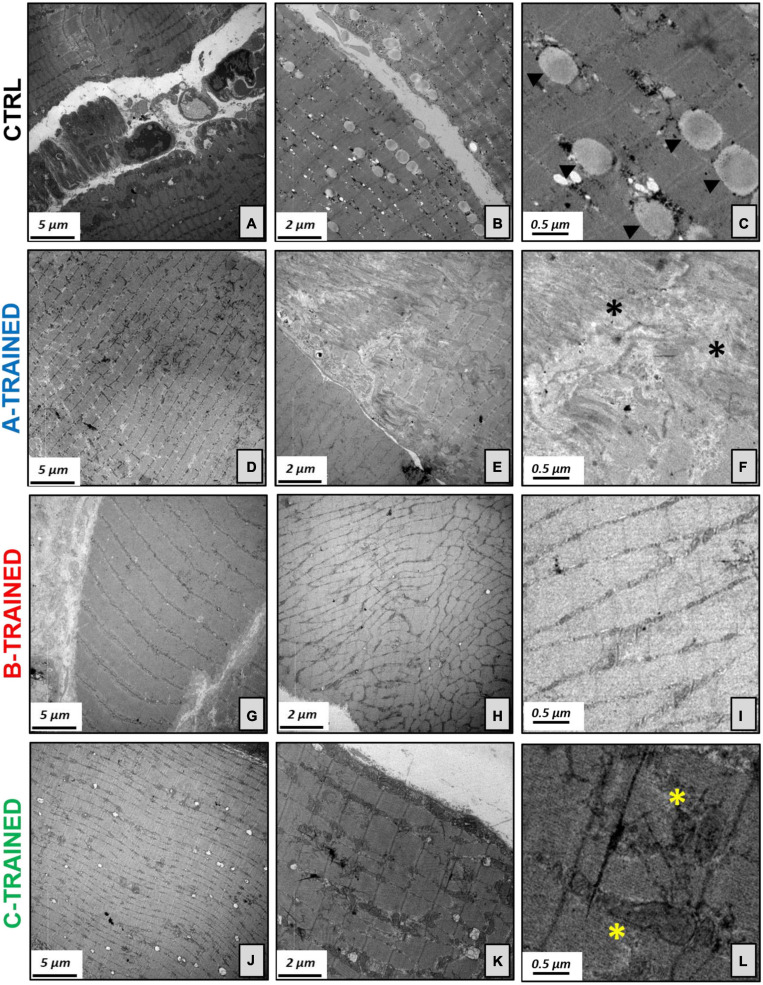
Electron microscopy analysis of the muscle tissues following WBV training. **(A)** Image displays degenerated sarcomeric network of CTRL groups, **(B)** with numerous lipid droplets and increase in atrophic fibers. **(C)** Electron micrograph shows several lipid droplets (arrowheads) next to mitochondria. **(D)** Muscle tissue of the A-TRAINED group was characterized by altered sarcomeric structures, **(E)** abundant inter-fiber fibrosis and presence of atrophic muscle fibers. **(F)** Enlargement of panel **(E)** highlights the fibrotic tissue (asterisks). **(G)** Image shows an improvement in muscle tissue fibers in the B-TRAINED group, **(H)** with the presence of normal fibers alternating with slightly atrophic ones. **(I)** Image displays a sarcomeric ultrastructure in a muscle of B-TRAINED mouse. **(J)** Muscle tissue of the C-TRAINED group shows normal sarcomeric structures, **(K)** with numerous and well-preserved mitochondria. **(L)** Enlargement of panel **(K)** highlights well-preserved dark mitochondria (asterisks) in a muscle of C-TRAINED mouse. Scale bar, 5, 2, and 0.5 μm.

These results were confirmed by the ultrastructural analysis of the muscle tissue performed by TEM and shown in Figure 3. Specifically, the muscle tissue of both control groups (Figures 3A–C) showed moderate misalignment of sarcomeric structures and the presence of numerous lipid droplets; in addition, we observed an increase in atrophic fibers and moderate fibrosis. Similarly, the muscle tissue of the A-TRAINED group (Figures 3D–F) was always characterized by altered sarcomeric structures, abundant inter-fiber fibrosis and a slight/moderate atrophy of the muscle fibers. In the muscle tissue of the B-TRAINED group (Figures 3G–I), we observed an improvement in cellular conditions, with the presence of normal fibers alternating with slightly atrophic fibers. Finally, mice trained with the C protocol (Figures 3J–L) did not present any significant alteration at the muscular level; on the contrary, we observed normal sarcomeric structures with numerous and well-preserved mitochondria.

## Discussion

In our previous work, we reported that exposure to an appropriate WBV protocol improves learning and memory processes in old mice (Cariati et al., 2021). These brain functions are particularly susceptible to the aging process. Indeed, it has been proposed that cognitive decline may occur as early as middle age, although the molecular mechanisms underlying the onset of the first typical symptoms of aging are not fully understood (Nacher et al., 2003; Shetty et al., 2005). Therefore, considering our previous results, in this work we examined the WBV impact on synaptic plasticity in a middle-aged murine model, also assessing whether this type of training can improve the decline in muscle function related to a sedentary lifestyle.

First, we performed *in vitro* extracellular recordings in hippocampal slices from 12-month-old mice to evaluate if the application of three WBV training protocols, differing in vibration frequency and vibration exposure time, had any effect on synaptic plasticity. We surprisingly observed that WBV training can counteract the LTP inhibition that characterized the control groups, although in a different way depending on the protocol used. Undoubtedly, we obtained better results for the groups trained with B and C protocols, in which the PS amplitude values, following a tetanic stimulus induced at the 15th minute of recording, remain significantly higher throughout the electrophysiological recording time, compared to the other experimental groups. In contrast, the A training protocol seems to modulate LTP in a biphasic manner, since we observed first a significant improvement (in the first 15 min of electrophysiological recording) and then a drastic reduction in synaptic plasticity (from about the 45th minute of recording), with PS amplitude values significantly different from those of the other experimental groups. These results are in agreement with previously published data (Cariati et al., 2021), suggesting that the A protocol, characterized by a high-vibration frequency (90 Hz) and a shorter recovery time (1 min between each series), is not suitable to be administered to either young, middle-aged or old mice, as it could be related to the onset of structural and functional alterations in the brain. In contrast, B and C protocols both positively modulate synaptic plasticity, suggesting that changing the protocol by even one parameter is sufficient to reverse the LTP inhibition observed in the control groups, which is known to be caused by a decrease in *N*-methyl-D-aspartate receptor activity that occurs during the aging process and is partially linked to a condition of oxidative stress (Tombaugh et al., 2002; Billard and Rouaud, 2007; Bodhinathan et al., 2010; Kumar, 2015; Kumar and Foster, 2019). As regard the different effects of WBV protocols on synaptic plasticity, it has been shown that mechanoreceptors, after receiving vibratory signals from different parts of the body, generate frequencies of action potentials that depend on the frequency and amplitude of the applied mechanical vibration, thus, driving the activated afferents to a discharge frequency identical to that of stimulation (Hagbarth and Eklund, 1968; Bosco et al., 1999b). Thus, we hypothesized that, depending on the used vibration frequency, a change in the analysis of proprioceptive information may occur, while the persistence of the effects could suggest the actual induction of plastic changes in the proprioceptive circuit (Cariati et al., 2021).

Surprisingly, the results obtained from the *in vitro* extracellular recordings in the hippocampal slices coincided perfectly with the data obtained from the histomorphometric and ultrastructural analysis of the muscle tissues by optic and transmission electron microscopy, respectively, with the aim of assessing whether WBV training could also influence on muscle plasticity. We observed that B and C training protocols induced, respectively, a partial and total recovery of the muscle morphological structure, characterized by an improvement in cellular conditions, correct sarcomeric organization, well-preserved mitochondria, little and/or no fibrosis and, more generally, an increase in the muscle fibers diameter compared with the control groups. About the A training protocol, ultrastructural analysis showed that the muscle tissue of the trained mice presents characteristics very similar to those of the muscle tissue of the control mice, with altered sarcomeric structures, abundant inter-fiber fibrosis and the presence of numerous atrophic fibers of reduced diameter. Again, we hypothesized that the A training protocol, due to the high vibration frequency and shorter recovery time, is not suitable for middle-aged mice, as the muscle structure is compromised in a similar way to that of control mice. Therefore, we believe that this WBV protocol has limited effectiveness in preventing the appearance of the first symptoms of the aging process and in counteracting the decline in muscle function typical of a sedentary lifestyle. These results allow us to argue that different patterns of muscle plasticity may evolve in response to different types of vibratory training, and that muscle plasticity may depend largely on the protocol used. This is demonstrated in the mice trained with the C protocol, characterized by a low-vibration frequency and a longer recovery time, that induced a total recovery of the muscle morphological structure, with no evidence of stress at sub-cellular level, compared with the other experimental groups.

Although our data suggest the use of WBV training as an effective strategy for improving cognitive and muscular function, doubts persist regarding its effects on other organs and tissues. It has been suggested that the repeated application of mechanical stimuli may increase the strength and stiffness of tendons through tissue hypertrophy (Kongsgaard et al., 2007; Couppé et al., 2008). However, the use of different WBV protocols makes it difficult to unambiguously determine the effects of vibration training on specific tissues. Indeed, low-intensity vibration appears to have no effect in joint tissues such as the Achilles tendon, patellar tendon, and medial collateral ligament; whereas, a higher vibration frequency may result in a significant weakening of the medial collateral ligament, but not other tissues (Keller et al., 2013). In addition, it has been reported that the human vestibulo-ocular system is extremely sensitive to vibrations, as it can be stimulated even by low vibration frequencies (Todd et al., 2008). However, the effects of a properly designed WBV protocol on this system have not been fully discussed. Therefore, further investigation is required to assess the impact of vibratory training on the entire body and not just on individual organs or systems.

## Conclusion

In accordance with the most recent scientific evidence, results obtained in our experimental study confirm the effectiveness of vibratory training in improving the ultrastructure and health of muscle tissue, as well as cognitive ability and, more generally, quality of life. Our data clearly indicate that the application of a WBV training protocol, appropriately designed in terms of vibration frequency and vibration exposure time, is appropriate and beneficial at both cerebral and muscular level. Noteworthy, different effects on synaptic and muscle plasticity were obtained by varying even the vibration frequency alone, suggesting the considerable impact of this parameter on the health status of mice. Indeed, exposure to high-frequency mechanical vibrations (90 Hz) resulted in significant damage in both brain and muscle, while partial and/or total recovery of age-related cognitive and muscular decline was found after the WBV training protocol application characterized by the same vibration exposure time, but by a lower vibration frequency (45 Hz). Surprisingly, the observed beneficial effects on synaptic and muscular plasticity are even greater after the WBV training protocol application characterized not only by a lower vibration frequency but also by a longer recovery time between one series and another, suggesting that the variation of this second parameter could be decisive for optimal physical performance. Therefore, these evidences indicate that the effects of WBV training depend not only on the vibration frequency and vibration exposure time but also on the recovery period between series, with an action comparable with a dose–response effect.

In conclusion, vibratory training, which has already proved to be an effective strategy to counteract the cognitive and motor decline typical of elderly subjects, can also represent an effective means of preventing and/or delaying the onset of the first symptoms typical of the aging process. Therefore, WBV can be used both to counteract the progression of age-related diseases, such as neurodegeneration and sarcopenia, and to reduce the deleterious effects of a sedentary lifestyle. Further studies will be necessary to determine the optimal WBV training protocol, also assessing the influence of parameters such as acceleration and displacement, which depend both on the vibration frequency and the intrinsic characteristics of different vibration platforms. In addition, it will be essential to understand the underlying mechanisms and investigate the presence of possible alterations in biological and molecular processes related to synaptic and muscle plasticity.

## Data Availability Statement

The raw data supporting the conclusions of this article will be made available by the authors, without undue reservation.

## Ethics Statement

All the experimental protocols were approved by the Italian Ministry of Public Health (authorization n. 86/2018-PR).

## Author Contributions

IC, RB, GA, GD, and VT developed the hypotheses and designed the experimental plan. IC, RB, and MS performed and analyzed the experiments, and contributing to the interpretation of the data. IC and RB wrote and edited the manuscript. GA, EB, GD, and VT assisted in drafts and final version of the manuscript. All authors read and approved the final manuscript.

## Conflict of Interest

The authors declare that the research was conducted in the absence of any commercial or financial relationships that could be construed as a potential conflict of interest.
